# Dr. Caius, of the Merry Wives of Windsor

**Published:** 1873-12

**Authors:** Jas. T. Whittaker


### DR. CAIUS, OF THE MERRY WIVES OF WINDSOR.

BY JAS. T. WHITTAKER, M.D.

When mystery was stripped from medicine, physicians looked and acted like other men. The practice of medicine in our day, being only the exercise of every-day principles in science, is a comparatively simple thing. The physician is an ordinary individual who has studied anatomy, chemistry, physiology, etc., instead of the various branches of law, or of letters, or of other science than medicine. Men of law, of letters, of science, understand him and respect him according to his actual acquisitions, or better, according to the stand which they give him in his profession. But the average physician of three centuries ago was a very different creature. He had, in the rule, no special information—in other words, no facts to stand on—so he worked with what is called tact. He adopted the plan suggested by Goethe, who was himself a physician, viz: to substitute for facts, phrases; for ideas, words. He found, in the credulity and superstition of the masses, fertile fields for the germination of this kind of grain, and he harvested in proportion. The nature of the soil has been so changed since, by the cultivation of a couple of hundred years, that such harvests have become much smaller. The detection of pretence of any kind does not absolutely require instruction in the special field in which it is practiced. It is easily and everywhere recognized by that worldly wisdom which finds its chief expression in what is called, in very commonplace phrase, "an acquaintance with human nature."

Dickens was prince in this kind of knowledge, the knowledge of every-day life. Shakespeare was king. Dickens fell upon the hypocrisy in the middle and lower walks of life; Shakespeare saw it everywhere. He unveiled it in the political potentates of the earth; he stripped it in the professions as well. "Shakespeare," wrote Dr. Johnson, "was, above all other writers, the poet who held up to his readers a faithful mirror of manners and life." The Roman senator, Menenius, shows the buffoonery of his nature, and the Danish usurper is drunken and despicable. The miserable cant that was in law and in theology, far more then than now, partly on account of him, he bared before the world, and the fraud and the wily knavery that was in medicine did not escape him. Hence Dr. Caius, in the "Merry Wives of Windsor."

I prefer to believe that this satire on the medical profession was written with a view of exposing the ignorance and trickery which characterized it in his day, rather than that it was launched at any particular individual. He pitched upon the name of Caius simply as a well-known cognomen of pedantry. Neither John Kaye ("Fui Caius" is his epitaph) nor Theodore Mayerne, the Caii of different commentators, were the Sebastians of his archery. The only traits they had in common with Shakspeare's Caius were a knowledge of French, and the rank and prerogative of physicians at the court. They were the foremost men of their times. John Kaye, or Key, as it is sometimes written, was Professor at Cambridge, which college he founded (the gates of Caius still hold their name); President of the Royal College of Physicians; body physician to Edward VI, Mary and Elizabeth; translator and editor of the works of Galen, Schreibonius, Largius, and other classical writers. He was one (Linacre was the other) of the "two phenixes of their profession in our nation." (Fuller by Bucknill.) Mayerne was body physician to two kings of France and two of England, "and left a name of great honor and wealth behind him." They were men of wide acquirements—that is, so far as classical medicine is concerned, for scientific medicine was unknown in their day—whose education had been enriched by travels in foreign lands; and, though one of them (Mayerne) had been disgracefully expelled by the bigoted College of France, they both succeeded to all the honors of the profession in the field of their labors. Shakespeare had too much respect for the medical profession, as will subsequently appear, to select as subjects of such gross caricature the leaders of their times.

If I should venture to express an opinion, then, upon this disputed point, it would be to the effect that the character is altogether fanciful. He had been ordered by Queen Elizabeth to prepare a play showing Falstaff in love. He had, according to authentic evidence, but fourteen days in which to elaborate his plot. Falstaff is the hero of the play; the other characters merely form the support, and the Doctor, with his murderous French, is introduced only as an offset to the Parson with his barbarous Welsh.

Shakespeare started out with the intention to make of the doctor a caricature as coarse as possible, and he never lost sight of this intention all the way through. In the first place he pictures him in love, and lets him sigh like a furnace, etc., in the style which might be imagined of such a creature. Then he repsents this love as totally unrequited, that a green and yellow melancholy may at last claim him for her own. In appearance and character he is a fit associate of Falstaff, Bardolph and Pistol. He is served by a tricky housewife. He is a foreigner, and of all foreigners the most detested, a Frenchman. He distorts and defaces the English language in a way as to keep him the subject of constant ridicule. Bucknill speaks of it in a congratulatory way, that "Dr. Caius plays no medical role."

Act I. Scene IV.—*A room in Dr. Caius' house. Enter Mrs. Quickly, the Doctor's housewife; Rugby, his servant, and Simple, the Servant of Slender.*

The housewife stations Rugby to watch for him while she and Simple prepare for a good time.

*Mrs. Quickly.* "What, John Rugby! I pray thee, go to the casement and see if you can see my master, Master Doctor Caius, coming. If he do, i' faith, and find anybody in the house, here will be an old abusing of God's patience and the king's English."

*Rugby.* "I'll go watch."

*Quickly.* "Go, and we'll have a posset for 't soon at night, in faith, at the latter end of a sea-coal fire. [And then the character of the doctor's servant.] "An honest, willing, kind fellow as ever servant shall come in the house withal, and, I warrant you, no tell-tale nor no breed-bate; his worst fault is, that he is given to prayer; he is something peevish that way; but nobody but has his fault; but let that pass."

A doctor's servant given to prayer recalls the strike of the doctors' drivers in Edinburg, last year. They struck for relief on Sunday, as it interfered with their devotions.

Precipitately enters Rugby.

"Out, alas! here comes my master."

*Mrs. Quickly.* "We shall all be shent. Run, in here, good young man; go into the closet. [Shuts him in.] He will not stay long. [And then, princess of hypocrisy that she is.] What, John Rugby! John! what, John, I say! Go, John, go inquire for my master. [And then, sweet solicitude.] I doubt he be not well, that he comes not home. [And then unconcernedly sings.] And down, down a down."

Enter Dr. Caius, a blustering bully.

"Vat is dat you sing? I do not like dese toys. Pray, you go and vetch me in my closet un *boitier verd;* a box, a green-a box; do intend what I speak? a green-a box."

The green box must have referred to the doctor's supply of medicines, which they were required to have with them always on state occasions.

The housewife is, of course, greatly relieved in being ordered to go the closet herself.

*Mrs. Quickly.* "Ay, forsooth, I'll go fetch it you." [Aside.] I am glad he went not himself; if he had found the young man, he would have been *horn*-mad."

Horn-mad is very expressive. It is the fury of marital jealousy that is meant, and the frequent reference to horns, the well-known ornament of betrayed husbands in Shakespeare's works, gives us a good idea of the laxity of morals in his day. "I'll be sworn," says Mrs. Quickly, in another place, "as my mother was the day I was born."

A synonymous appelation to horns, and a stigma of the deepest dye applied to the husband of a faithless wife, was cuckold. "Hang him, poor cuckoldy knave," soliloquizes Falstaff over Ford; and, farther on, "I will use her as the key of the cuckoldy rogue's coffer." Again, "It (his cudgel) shall hang over him like a meteor o'er the cuckold's horn." Ford stigmatizes himself with, "Fie, fie, fie, cuckold! cuckold! cuckold!" The horns and the cuckold are expressive of the same meaning. Falstaff speaks of Ford, in another place, as a peaking *cornuto.* The last bone of the spinal column is called the coccyx, from the Greek word kokkuk a cuckoo, an "appelation assigned to that unnatural bird on account of the amorous and familiar coy which not ungratefully salutes our ears about spring-time of year." The note of the cuckoo is a call to love, and he sits on the bare branch of a tree repeating his song, which he loses when the amorous season is over. "This note is so uniform that the name seems to have been derived from it, and thus we have in the English, cuckoo; in the French, cou-cou; in German, kuckuk; in Italian, cuculo; Greek, kokko." The beak or horn of the cuckoo is black, strong, and curved. Dr. Jenner, who wrote a paper on the connection between the cuckoo, the horns and adultery, in the Philosophical Transactions, remarks that the Romans, observing their habits, applied the word cuculus, cuckoo, as a term of reproach; and then to the adulterous husband, because he acted like the cuckoo, who drops his eggs in another bird's nest, whilst the victimized husband was called cucurra, or hedge-sparrow, the bird whose nest was often selected by the cuckoo for the residence of its young, and who, in happy ignorance, educated the supposititious brood.

With the Grecian gods adultery was no uncommon recreation, and Jupiter is said to have transformed himself into a cuckoo in order to secure the affection of Juno. It is remarked as very singular, by Dr. Rivington, that married men, whose wives have been unfaithful, should have been popularly invested with horns, and that the term horns has been applied to the processes projecting upward from the coccyx. He was not able to find any other explanation for the origin of horns as the badge of cuckoldism. It has been quite learnedly discussed, he says, in many works, without definite result.

By horn-mad, then, it was meant that the doctor, possessed the temper of a husband infuriated by jealousy, the green-eyed monster, etc.

Now, it is barely possible that Mrs. Quickly meant the expression more literally. She boasts of her usefulness to the doctor. She bakes for him, sews for him, etc., in fact, does everything for him; kept his house, as she says herself; but from a careful reading of the whole text, I am not inclined to believe that Shakespeare wished to add this sin to the doctor's list.

To return to the doctor:

*Caius.* "Fe, fe, fe, fe, ma foi, il fait fort chaud. Je m'en vais a la cour—la grand affair."

In English, it was court day, and the doctor must go in state.

"Vere is dat knave, Rugby?"

"Here, sir."

"Come, take-a your rapier, and come after my heel to the court."

The doctor must have been in his court-dress, cue, wig and all. He had only to mount his horse, while his servant followed on foot behind. The ancient cane was a relic of the paraphernalia of the conjurer, the wand of magic. "It had a gold knob on the end, which was hollow, and contained a vinaigrette, which the doctor held to his nose when he approached a sick person, so that its fumes might protect him from the noxious exhalations of his patient."

Next to the cane, the wig. Will Atkins, the gout doctor, in

Charles II time, wore one with three tails. He never wore a hat for fear of disarranging it. One of the most magnificent wigs on record was that of Dalmahoy.

> "If you would see a noble wig,
> And in that wig a man look big,
> To Ludgate hill repair, my joy,
> And gaze on Doctor Dalmahoy."

And then there were the silk stockings, the silk coat, and lastly, the muff, "in which the fingers were held in cold weather, to keep them soft and delicate for nice observations on milady's pulse."

Aubrey says of the immortal Harvey, after a personal description, that he "rode a horseback with a foot-cloth to visit his patients, his man following on foot, as the fashion then was."

But the time flies, and the hour for court is at hand—suddenly:

*Caius.* "By my trot, I tarry too long. Od's me! Qu-ai-j'oublie! dere is some simples in my closet dat I vill not for the vorld I leave behind."

Precious stuff were these simples: Pulverized human bones, raspings of a skull unburied, balsam of bats, as an ointment for hypochondriacs, containing adders, bats, sucking whelps, earthworms, hog's grease, the marrow of an ox and the thigh bone of an ox.

In unlocking his closet for his simples, Caius discovers Simple and exhibits his choler. But this is only a touch of his real nature.

*Mrs. Quickly.* "I am glad he is so quiet; if he had been thoroughly moved you should have heard him so loud and so melancholy, etc.

A little later we are treated to an exhibition of this kind.

*Caius.* "You, Jack'nape; give a dis letter to sir Hugh; by gar it is a challenge; I will cut his troat in de park; and I will teach a scurvy jack-a-nape priest to meddle or make—you may be gone [he feels himself dangerous] it is not good you tarry here—by gar I will cut all his two stones, etc.

Act II. Scene III.—The Appointment for the Duel.

*Windsor Park.*—Enter Caius and Rugby.

*Caius.* "Jack Rugby!"

"Sir?"

"Vat is de clock, Jack?"

"Tis past the hour, sir, that Sir Hugh promised to meet."

*Caius.* "By gar, he has save his soul, dat he is no come; he has pray his Pible well, dat he is no come; by gar, Jack Rugby, he is dead already, if he be come."

*Rugby.* "He is wise, sir; he knew your worship would kill him, if he came."

"By gar, de herring is no dead so as I vill kill him. Take your rapier, Jack; I vill tell you how I vill kill him."

"Alas, sir, I cannot fence."

"Villany, take your rapier."

Fortunately for poor Jack, the dramatis personæ is suddenly increased at this juncture by the arrival of the rest of his company.

*Caius.* "Vat be all you, one, two, tree, four, come for?"

*Host.* To see the fight, to see the foin, to see thee traverse; to see thee here, to see thee there. Is he dead, my Ethiopian? is he dead, my Francisco? ha, bully! What says my Æsculapius? my Galen? my heart of elder? ha! is he dead, bully Stale? is he dead?"

*Caius.* [foamingly indignant] "By gar, he is de coward Jack priest of de vorld; he is not show his face."

*Host.* [continues his expression of esteem] "Thou art a Castalian-King-Urinal! Hector of Greece, my boy!"

The epithet "urinal" has reference to the pretended analysis of urine, then the fashion, and still by some charlatans relied on exclusively in establishing a diagnosis.

*Caius.* "I pray you, bear vitness that me have stay six or seven, two, tree hours for him, and he is no come."

*Shallow.* "He is the wiser man, master doctor; he is a curer of souls, and you a curer of bodies; if you should fight, you go against the hair of your professions. Is it not true, Master Page?"

They amuse themselves awhile over the doctors English, when the Host finally cools him down by promising to bring him to the object of his affections.

The doctor is not insensible to this kindness and promises requital, as follows:

"By gar, me tank you far dat; by gar; I love you and I shall procure-a you de good guest, de earl, de knight, de lords, de gentlemen, my patients."

"For the which," [replies the host, deeply touched, no doubt], "I will be thy adversary towards Anne Page."

Now the preacher, the other combatant, who is waiting at a distant place according to the plot, grows alike impatient, but his impatience is not exactly of the same order. In fact he rather congratulates himself on the present status of things."

*Evans.* "I pray you now, good master Slender's serving-man, and friend Simple by your name, which way have you looked for master Caius, who calls himself Doctor of Physic?"

*Simple.* "Marry, sir, the city ward, the park ward, every way but the town way."

*Evans.* "I most fehementle desire, you will also look that way."

"Pless my soul, how full of cholers I am, and trempling, of mind! I shall be glad if he have deceived me—how melancholies I am," etc.

How full of cholers I am, how melancholies, etc.

Popular opinion connects bile and bad temper and melancholy together perhaps more closely, observes Tuke, than any other physical and psychical facts. It is said of a man thus that he displays a great deal of bile and from the same cause originates the word choleric. Homer speaks of the cholos of Achilles in his Iliad. "Achilles bears no gall within his breast" and it may be noted as marking the interchangeableness of bodily and mental terms that it is as fitting, to speak of the "gall of bitterness" as of the "bitterness of gall."

The Latin poets abound with references to the connection between the liver and the emotions, as in Juvenal "*Quid referan—quanta siccum jecur ardeat ira.* But such passages bear more especially upon the supposed seat of anger in this organ. But it was not anger alone which was supposed to be connected with the liver. The jecur ulcerosum of Horace was induced by love. Pistol tells us that Falstaff loves his (Ford's) wife with Liver burning hot. Plautus termed this feeling morbus hepatarius; Solomon speaks of the misguided youth in whom the dart of passions strikes through his liver. It is to grief that Jeremiah alludes when he complains "my liver is poured upon the earth."

Another instance in this play of reference of a psychical function to a physical organ is contained in Falstaff's allusion to the kidneys as the seat of lust:

*Falstaff.* "Come let me pour some sack to the Thames water; for my belly 's as cold as if I had swallowed snowballs for pills to cool the veins.

But to return to the field of battles, we left the parson awaiting the doctor in that state of mind which is said to characterise the soldier when drawn up in battle array awaiting his unseen foe. He takes this opportunity, however, to express his opinion about the doctor.

*Enter Page and Shallow.—Page.* "We are come to you to do a good office, master parson."

"Ferry well; what is it?"

*Page.* "Yonder is the most reverend gentleman, who, belike having received wrong by some person, is at most odds with his own gravity and patience that ever you saw."

*Shallow.* "I have lived fourscore years and upward; I never heard of a man of his place, gravity and learning, so wide of his own respect."

[The most ignorant pretender thus has his supporters.]

*Evans.* "What is he?"

*Page.* "I think you know him; Master Doctor Caius, the renowned French physician."

*Evans.* Got's will, and his passion of my heart! I had as lief you would tell me of a mess of porridge."

"Why?"

*Evans.* "He has no more knowledge in Hibocrates and Galen —and he is a knave besides; a cowardly knave as you would desires to be acquainted withal."

The doctor now appears with mine host and his crowd of lunch fiends.

The doctor and the parson entertain each other with the vilest expletives in the vilest Welsh and French-English, when mine host steps in between them and brings about a reconciliation in a manner which should forever secure him that blessing pronounced upon the makers of peace.

*Host.* Peace, I say! hear mine host of the Garter. Am I politic? am I subtle? am I a Machiavel? Shall I lose my doctor? no; he gives me the potions and the motions. Shall I lose my parson, my priest, my Sir Hugh? no; he gives me the proverbs and the no-verbs. Give me thy *hand*, terrestrial; so. Give me *thy* hand, celestial; so. Boys of art, I have deceived you both; I have directed you to wrong places; your hearts are mighty, your skins are whole, and let burnt sack be the issue."

And thus ended the doctor's fight.

And now it is Anne's turn to vilify the luckless doctor. Her mother tells her she has sought her a better husband than yon fool, the parson.

[An instance of the proverbial power of doctors with old women.]

*Mrs. Quickly.* "That's my master, the doctor."

*Anne.* "Alas, I had rather be set quick i' the earth. And bowl'd to death with turnips!"

And last of all it is Mrs. Quickly's turn. She boasts to Fenton the accepted lover, that she had said to Anne's mother "Nay, will you cast away your child on a fool, and a physician?"

A fool and a physician.

Even as late as the 15th century, an apprentice would not be accepted in any of the mechanical arts unless he could prove that he was not the child of a butcher, an executioner or a bleeder.

The following old-time advertisement clipped from a paper of Shakespeare's day is curious and instructive:

"Wanted.—In a family who have had bad health, a sober, steady person in the capacity of doctor, surgeon, apothecary and man midwife. He must occasionally act as butler and dress hair and wigs. He will be required sometimes to read prayers and to preach a sermon every Sunday. A good salary will be given."

The rest of Dr. Caius is a short story. He is duped at Herne's Oak in Windsor Park and made to carry off a boy in lieu of his lady-love. He rushes frantically upon the scene with:

"Vere is mistress Page? By gar, I am cozened. I ha' married un garçon, a boy; un paysan, by gar, a boy; it is not Anne Page, by gar, I am cozened. By gar, I'll raise all Windsor." *Exit Caius.*

The ridiculous role assigned to the physician in this play, together with Macbeth's petulant order to his physician:

"Throw physic to the dogs, I'll none of it." (Act V—Scene 3.)

and the sad picture of poverty and venality in the sketch of the apothecary:

* * * "famine is in thy cheeks,
Need and oppression stareth in thy eyes,
Upon thy back hangs ragged misery,
The world is not thy friend, nor the world's law," etc.

[Romeo and Juliet, Act V, Scene 1.]

have furnished material for many bitter reflections against the profession of medicine, and have been quoted in derision of it from time to time by the feeble imitators of Moliere.

But the truth is, The Merry Wives of Windsor is the only play of Shakespeare in which a physician is held up to ridicule. (Pinch, in the comedy of Errors, is a schoolmaster and a conjurer, not a physician.) Macbeth's injunction was an expression of impatience on being told that for a mind diseased and rooted sorrow "Therein the patient must minister to himself." The apothecaries of Shakespeare's day were the bitterest enemies of the physicians, and the description as above given would have been applauded to the echo by the doctor's themselves.

Medical references are so abundant in all Shakespeare's works (with the single exception of Titus Andronicus) and the allusions are so classical as to have given rise to the opinion that Shakespeare absolutely studied medicine himself, perhaps even practiced it. I find this opinion discussed in detail in Bucknill's Medical Knowledge of Shakespeare, London, 1860; Cless' Medicinische Blumenlese aus Shakespeare, Stuttgart, 1865, and Shakespeare als Mediziner, by Dr. Hermann Aubert, Prof. of Physiology at Rostock, Rostock, 1873. None of these commentators, however, claim Shakespeare as a physician, but the testimony is universal that he must have been intimate with the physicians of his time, and that he must have "devoured" the medical, just as he did every other variety of literature of his day. Take the following as one of many technical terms or phrases used in different plays, whose expression on a railway train, for instance, or at a table d'hote, would at once, as Aubert suggests, convey the impression to a professional man that the speaker was a physician. "These are begot in the ventricle of memory, nourished in the womb of pia mater" (Love's Labor Lost. Act II.) See also Ray's portrayal of insanity by Shakespeare, Amer. Journ. Insanity, Vol. III, with a review of the same by Cless in the Allgem. Zeitschrift fur Psychologie VI, p. 174; Lear and Ophelia, a lecture by Prof. Heinrich Neumann, Breslau, 1866; "Kenig Lear," eine psychologische Shakespearestudie, by Dr. Carl Stark, Stuttgart, 1871.

Shakespeare had the opportunity of an intimate association with a physician at his own hearthstone. His eldest daughter, Susanna, married Dr. John Hall, "a physician of great provincial eminence" at Stratford upon Avon. Dr. Hall subsequently became "joint occupier" of Shakespeare's house, and treated him, it is supposed, during several attacks of illness.

It has been well said of Shakespeare's works that they are but pictures of the every-day life of his time. The very language he uses was employed by those with whom he was in constant intercourse. Some, much, indeed, of this association was with physicians. It would be absurd to claim that he was able to read the mysteries of science which have only since been discovered by the labors of subsequent centuries, and thus see the absurdities in the practice of even the most eminent physicians of his day. It would be absurd to think that he would rail against men equally shrewd with himself in worldly wisdom and superior to him often in scholastic lore. At the most he could only understand and appreciate such characters at their true worth, and how he succeeded in this regard we learn from the parts which physicians are made to play in his other works.

"The older physician therefore, was a man of the world, whose good manners, formed by travel and learning, provided a decent cloak for his scientific ignorance, and whose good sense and keen observation enabled him to look into the secrets of men, when those of nature were still unrevealed. This is the "kind of men" of which a physician ought to be, according to Queen Elizabeth, as we see in her letter recommending her physician, Dr. James, to the Emperor of Russia: "Quod hominum genus (medicum) quoniam et plurimarum rerum cognitionem et morum probitatem non vulgarem postulat." Such physicians Shakespeare may have known in Dr. James, or Kaye, or Harvey, and certainly did know in his son-in-law Dr. Hall. Such physicians he has represented in Lear, Cymbeline and Macbeth." (Bucknill.) *The Clinic.*

